# Body Dissatisfaction, Importance of Appearance, and Body Appreciation in Men and Women Over the Lifespan

**DOI:** 10.3389/fpsyt.2019.00864

**Published:** 2019-12-17

**Authors:** Hannah L. Quittkat, Andrea S. Hartmann, Rainer Düsing, Ulrike Buhlmann, Silja Vocks

**Affiliations:** ^1^Department of Clinical Psychology and Psychotherapy, Institute of Psychology, Osnabrück University, Osnabrück, Germany; ^2^Department of Research Methodology, Diagnostics & Evaluation, Institute of Psychology, Osnabrück University, Osnabrück, Germany; ^3^Clinical Psychology and Psychotherapy, Institute of Psychology, University of Münster, Münster, Germany

**Keywords:** body image, body dissatisfaction, body appreciation, importance of appearance, gender comparison, age, lifespan

## Abstract

Body image disturbance is associated with several mental disorders. Previous research on body image has focused mostly on women, largely neglecting body image in men. Moreover, only a small number of studies have conducted gender comparisons of body image over the lifespan and included participants aged 50 years and older. With regard to measurement, body image has often been assessed only in terms of body dissatisfaction, disregarding further aspects such as body appreciation or the importance of appearance. The aim of this cross-sectional study was to explore different aspects of body image in the general German-speaking population and to compare men and women of various ages. Participants completed an online survey comprising questionnaires about body image. Body dissatisfaction, importance of appearance, the number of hours per day participants would invest and the number of years they would sacrifice to achieve their ideal appearance, and body appreciation were assessed and analyzed with respect to gender and age differences. We hypothesized that body dissatisfaction and importance of appearance would be higher in women than in men, that body dissatisfaction would remain stable across age in women, and that importance of appearance would be lower in older women compared to younger women. Body appreciation was predicted to be higher in men than in women. General and generalized linear models were used to examine the impact of age and gender. In line with our hypotheses, body dissatisfaction was higher in women than in men and was unaffected by age in women, and importance of appearance was higher in women than in men. However, only in men did age predict a lower level of the importance of appearance. Compared to men, women stated that they would invest more hours of their lives to achieve their ideal appearance. For both genders, age was a predictor of the number of years participants would sacrifice to achieve their ideal appearance. Contrary to our assumption, body appreciation improved and was higher in women across all ages than in men. The results seem to suggest that men’s and women’s body image are dissimilar and appear to vary across different ages.

## Introduction

Many people are concerned about at least one part of their body (1). A negative cognitive evaluation of one’s body can be an expression of a negative body image (2). Body image is conceptualized as a multidimensional construct, which encompasses a behavioral component involving body-related behaviors (e.g. checking behaviors), a perceptual component involving the perception of body characteristics (e.g. estimation of one’s body size or weight), and a cognitive-affective component involving cognitions, attitudes, and feelings toward one’s body (3–6).

Negative thoughts and feelings about one’s body are defined as body dissatisfaction (7), which is considered to be the most important global measure of stress related to the body (4). Body dissatisfaction has been found to be a predictor for the development of an eating disorder (8) and occurs in individuals with different mental disorders, such as binge eating disorder or social anxiety disorder (e.g. 6, 9), as well as in healthy persons (e.g. 10–12). It represents one of the two poles of the satisfaction-dissatisfaction continuum of body image disturbance (4), which encompasses measures of satisfaction (e.g. being satisfied with particular body areas; e.g. 13) and dissatisfaction (e.g. weight or muscle dissatisfaction; e.g. 14, 15).

Another construct which is related to both the cognitive-affective and the behavioral component is the importance of appearance, also termed appearance orientation, which reflects the cognitive-behavioral investment in one’s appearance as an expression of the importance people place on their appearance (16, 17). This construct was shown to be distinguishable from the construct of appearance evaluation (18), which also represents a measure of body satisfaction/dissatisfaction.

Besides negative body evaluation and the importance of appearance, a positive appraisal of one’s body also forms part of the cognitive-affective component. For instance, body appreciation is defined as accepting, respecting, and having a favorable opinion of one’s own body, as well as rejecting unrealistic body ideals portrayed by the media (19). Body appreciation was shown to predict indices of well-being beyond other measures of body image (19) and occurred simultaneously with body dissatisfaction, highlighting the independence of the two concepts (20).

In the past, studies have investigated the impact of gender and age on body features related to the cognitive-affective component. Specifically, research on body dissatisfaction has shown that girls and female adolescents (e.g. 21–24), and women of all ages (e.g. 12, 25, 26) report body dissatisfaction. While some studies revealed that the level of body dissatisfaction varied across different age groups (27, 28), others found that body dissatisfaction remained quite stable across the adult lifespan in females (20, 25, 29, 30). Studies examining other aspects of the satisfaction-dissatisfaction continuum, such as weight dissatisfaction (15, 31) or satisfaction with particular body parts (13, 32), also found body dissatisfaction in women. Frederick and colleagues (33) estimated that 20% to 40% of women are dissatisfied with their bodies. Nevertheless, body dissatisfaction is also reported in men, suggesting that 10% to 30% of men show body dissatisfaction (33) or 69% of male adolescents to be dissatisfied with their bodies in terms of their weight (34). Frederick and colleagues (14) even reported that 90% of male US students in their sample described themselves as being dissatisfied with respect to muscularity. In terms of body evaluation, striving for increased muscularity, referred to as drive for muscularity (35), has emerged as a central issue for boys and men (e.g. 35–38). It was shown to be distinct from body dissatisfaction (39). However, although previous studies reported that body dissatisfaction does not differ across age in women, it remains unclear whether the level of body dissatisfaction changes across age in men.

While body dissatisfaction seems to remain stable across age in women, studies suggest that the importance of appearance appears to decrease with age (40). In line with Pliner and colleagues, Tiggemann and Lynch (41) found in a group of females aged 20 to 84 years that the importance of appearance was lower in older than in younger women. For men, only one study has examined the importance of appearance, and found that it varied between age groups and reached a peak at age 75 years and older (42). To our knowledge, no other study has examined the importance of appearance in men over the lifetime. Thus, it remains relatively unclear whether the importance of appearance remains stable or changes over the lifetime in men.

With respect to body appreciation, Tiggemann and McCourt (20) demonstrated higher body appreciation in older than in younger women. Furthermore, high body appreciation was found to be protective against the negative effects of media exposure to thin models in women (43). Other studies reported that body appreciation in men and women was associated with a low level of consumption of Western and appearance-focused media (44) and correlated negatively with internalization of sociocultural ideals (45). However, studies focusing on age differences regarding body appreciation in males are lacking.

Previous studies on body image have mostly considered age-related changes in either men or women, or in particular age groups (e.g. college students, adolescents). Only a limited number of studies have compared men and women with respect to the aforementioned aspects of body image. These studies generally found greater body dissatisfaction in females than in males (e.g. 29, 30, 46–49). Men (vs. women) seem to place less importance on their appearance (42, 50, 51) and report slightly higher levels of body appreciation (e.g. 45, 52–54). Tylka and Wood-Barcalow (55) also reported higher body appreciation in college men (vs. college women), but were unable to replicate this effect in a community sample. In contrast to this latter result, Swami and colleagues (53) reported higher body appreciation in men than in women in a sample from the general Austrian population. However, these studies comparing men and women did not analyze their data with respect to the impact of age.

Only a small number of studies have investigated the effect of age and gender on body dissatisfaction, importance of appearance and body appreciation. In a two-year longitudinal study, Mellor and colleagues (56) found that body dissatisfaction was higher in females than in males and higher in younger than in older participants. In another longitudinal study, Keel and colleagues (15) examined men and women over a period of 20 years. As men aged, the authors observed increasing weight and increasing weight dissatisfaction, while weight dissatisfaction decreased in women despite analogous increases in weight. The authors concluded that women appear to be more accepting of their weight as they age (15). Unfortunately, the mean age at the 20-year follow-up was only 40 years, meaning that conclusions could not be drawn about the whole adult lifespan. Similarly, in a large sample of men and women aged 18 to 49 years, Ålgars et al. (46) found that overall body dissatisfaction was higher in women than in men, but that only in women was age associated with decreasing body dissatisfaction, while in men, body dissatisfaction changed across the different age groups (46). However, these results have to be interpreted with caution, as the sample consisted of twins and was thus not representative of the general population.

Other studies found higher levels of body dissatisfaction (28) and lower levels of satisfaction with certain body areas (29) in women than in men. However, the latter study did not find any gender- or age-related effect on overall body dissatisfaction (29). Concerning the importance of appearance, Öberg and Tornstam (42) found that women placed more importance on their appearance than did men, and that this factor remained stable across different age groups in women but varied in men. These results are contrary to the findings of Tiggemann and Lynch (41) and Pliner et al. (40), who found that the importance of appearance decreased with age in women. However, this discrepancy may be due to the assessment method in the study by Öberg and Tornstam, as they used a single item to evaluate the importance of appearance. Hence, the development of importance of appearance in men and women across the lifespan remains unclear.

Although, as mentioned above, some studies have found that women place less importance on their appearance as they age (40, 41), this aspect has not been examined in a large population sample comprising different age groups in relation to the impact of gender and age. Furthermore, studies comparing body appreciation between men and women across different age groups are lacking. To our knowledge, no previous study has examined body dissatisfaction, importance of appearance and body appreciation in the general population including men and women aged 16 to 50 years and older. Therefore, the present study aims to fill this research gap by analyzing these negative and positive aspects of body image in a general population sample considering gender and age.

First, based on the previous findings outlined above, we predicted that body dissatisfaction would be higher in women than in men (Hypothesis 1) and would remain stable across age in women (Hypothesis 2). As no previous study has investigated body dissatisfaction across the whole lifespan in men, we aimed to examine a potential influence of age on body dissatisfaction in men.

Second, we hypothesized that women would place more importance on their appearance than men (Hypothesis 3), but that in line with the aforementioned studies, across age, older women would report lower levels of importance than younger women (Hypothesis 4). Given the lack of corresponding studies in men, we intended to investigate the importance of appearance and its relation to age in men in an exploratory analysis. Furthermore, appearance orientation assesses the importance of appearance in terms of the extent of investment in one’s appearance (e.g. grooming behaviors) and in terms of the attention one pays to one’s appearance. However, it does not quantify how many hours or years people would be willing to invest in their appearance to look the way they want to. Therefore, as a measure of the importance of appearance, we additionally assessed the number of hours men and women would be willing to invest per day to achieve their ideal appearance, and the number of years of their life they would sacrifice to achieve their ideal appearance.

Third, we predicted that body appreciation would be higher in men than in women (Hypothesis 5). As the aforementioned studies examined gender differences without analyzing the impact of age, we aimed to investigate potential changes in body appreciation across age in an exploratory manner.

Fourth, to take into account the well-documented increase in BMI over the lifetime (e.g. 46, 57, 58) and its potential association with the outcome variables, we examined these relations as a control analysis by calculating correlations between the subjective evaluations of body image and BMI.

## Materials and Methods

### Participants

Inclusion criteria were age 16 years and older, sufficient German-language skills, and internet access. Data were collected from *N* = 1,338 persons. From the original data set, *n* = 4 participants had to be excluded due to ambiguous details about their age or invalid responses to questions. Moreover, *n* = 7 persons were excluded as they did not fit into the binary gender categories male or female. The final study sample comprised *n* = 942 women and *n* = 385 men, aged 16 to 88 years (total sample: *n* = 1,327).

### Measures

#### Demographic Data

All participants completed a questionnaire assessing demographic data such as gender, age, height and weight, educational level, relationship status, sexual orientation, and number of children. The item on sexual orientation was optional. Self-reported weight and height were used to calculate the body mass index (BMI, kg/m^2^).

#### Multidimensional Body-Self Relations Questionnaire–Appearance Scales

The Multidimensional Body-Self Relations Questionnaire–Appearance Scales [MBSRQ-AS; (16); German-language version: (17)] is a self-report questionnaire consisting of 34 items and five subscales to assess different appearance-related aspects of body image. The MBSRQ-AS has been validated for participants aged 15 years and older and for both men and women (16). For the purpose of this study, the *Appearance Evaluation Scale* (seven items) and *Body Areas Satisfaction Scale* (nine items) were used to assess body dissatisfaction, and the *Appearance Orientation Scale* (12 items) was applied to examine the importance people place on their appearance. According to Cash (16), the *Appearance Evaluation Scale* measures overall satisfaction/dissatisfaction with one’s appearance and physical attractiveness, with high scores indicating body satisfaction and low scores indicating body dissatisfaction. Furthermore, the *Body Areas Satisfaction Scale* (nine items) assesses satisfaction/dissatisfaction with particular body areas; high and low scores are analogous to the Appearance Evaluation Scale. The *Appearance Orientation Scale* (12 items) evaluates the investment in one’s appearance, with low scores indicating that people do not place importance on or invest much effort into being “good-looking”. All items are rated on a 5-point Likert scale with different response labeling (*Appearance Evaluation Scale* and *Appearance Orientation Scale*: 1 = definitely disagree to 5 = definitely agree; *Body Areas Satisfaction Scale*: 1 = very dissatisfied to 5 = very satisfied). While the English-language version has been validated in both men and women (16), the German-language version has only been validated for females (17). In the German validation, all subscales showed good internal consistency (α = .78–.90; 17). In the current sample, high internal consistencies were found (*Appearance Evaluation Scale*: α = .88; *Appearance Orientation Scale*: α = .85; *Body Areas Satisfaction Scale*: α = .81), both for men (*Appearance Evaluation Scale*: α = .87; *Appearance Orientation Scale*: α = .85; *Body Areas Satisfaction Scale*: α = .80) and women (*Appearance Evaluation Scale*: α = .89; *Appearance Orientation Scale*: α = .86; *Body Areas Satisfaction Scale*: α = .81).

#### Body Appreciation Scale-2

The Body Appreciation Scale-2 (BAS-2; 55; German-language version: Steinfeld, unpublished manuscript) assesses body appreciation in a gender-neutral manner using 10 items rated on a 5-point Likert scale (1 = never to 5 = always). High internal consistency (α = .96) was found for the BAS-2 in an English-speaking sample of men and women (55). In our sample, internal consistency was high (α = .94), both in males (α = .92) and females (α = .94).

#### Investment in One’s Appearance

To investigate the amount of time which men and women would be willing to invest in and sacrifice for their own appearance, participants were asked the following two questions: “How many years of your life would you be willing to sacrifice if you could look the way you want?”, “How many hours a day would you invest in your appearance if you could look the way you want?”

#### Single-Item Self-Esteem Scale

The Single-Item Self-Esteem Scale (SISE; 59) measures self-esteem using the item “I have high self-esteem,” which is rated on a 5-point Likert scale (1 = not very true of me to 5 = very true of me). It has shown high correlations with the Rosenberg Self-Esteem Scale and a high test-retest reliability after four years (*r* *_tt_* = .75) (59).

#### Depression Anxiety Stress Scales–Depression Subscale

The Depression Anxiety Stress Scales–Depression Subscale (DASS-D) (60; German-language version: 61) consists of seven items assessing depressive mood over the past week on a 4-point Likert scale (0 = never to 3 = always). For the German version of the DASS-D, high internal consistency has been found (α = .88) (61). In the present study, internal consistency ranged from α = .89 for men to α = .91 for women (total sample: α = .90).

### Study Procedure

Participants were recruited via social media, mailing lists, press releases, advertisements, and flyers and were asked to take part in a short online survey comprising different questionnaires about body image. To access the study website, they could either scan a barcode or use a web link. The online survey was set up using the software Unipark (Version EFS Winter 2018; 62). Participants were informed about the purpose of the study and were asked to provide their informed consent by clicking a button next to a declaration asserting that they agree to the processing of their personal data according to the given information. The survey began once participants had provided consent and took approximately 10 min to complete. Participants were offered no financial compensation for study participation. The research project was conducted in accordance with the Declaration of Helsinki and was approved by the ethics committee of Osnabrück University.

### Data Analysis

Data analysis was performed using the software SPSS Statistics (version 25; IBM 63) for descriptive statistics, correlation analysis, and general linear models and the software R (version 3.5.3; R 64) with the DHARMa package (version 0.2.4; 65), the glmmTMB package (version 0.2.3; 66), and the MASS package (version 7.3–51.3; 67) for generalized linear models. As we intended to explore homogenous hypotheses in terms of body dissatisfaction, the power was set at a significance level of *p* = .10 for the variable age.

For group comparisons on demographic and descriptive variables (**Table 1**), we calculated Mann-Whitney U Tests, as our data were not normally distributed (except BMI). Since inferential statistics for simple comparisons are massively overpowered in such large samples, we additionally report effect sizes. For better interpretability, *U*-values were converted into correlation coefficients *r* (68, 69). For correlations between BMI and the body image variables (**Table 3**), Spearman’s rank correlations were calculated due to non-normally distributed data.

**Table 1:** Descriptive statistics and group comparisons regarding age, height, weight, BMI, depression, and self-esteem.

	Total sample			Women			Men			Test Statistics		
	*M (SD)*	*N*	Min; Max	*M (SD)*	*N*	Min; Max	*M (SD)*	*N*	Min; Max	*U*	*p*	*r*
Age	33.05 (14.09)	1,327	16; 88	31.40 (13.33)	942	16; 83	37.08 (15.08)	385	16; 88	134,235	***	0.2
Height	173.01 (9.04)	1,322	150; 212	169.15 (6.46)	938	150; 188	182.45 (7.33)	384	160; 212	31,581.5	***	0.65
Weight	72.61 (16.89)	1,319	40; 175^+^	67.73 (14.75)	936	40; 155	84.55 (15.85)	383	55; 175^+^	63,599	***	0.51
DASS-D	1.60 (0.62)	1,319	1; 4	1.62 (0.64)	937	1; 4	1.56 (0.57)	382	1; 4	187,904.50	n.s.	0.04
SISE	2.84 (0.76)	1,327	1; 4	2.77 (0.76)	942	1; 4	3.01 (0.72)	385	1; 4	151,165.50	***	0.14
										***T***	***p***	***r***
BMI	24.15 (4.88)	1,314	15.43; 62.75^+^	23.65 (4.93)	932	15.43; 49.01	25.37 (4.55)	382	17.32; 62.75^+^	5.88	***	0.18

Age in years; height in centimeters; weight in kilograms. DASS, Depression Anxiety Stress Scale–Depression Subscale; SISE, Single-Item Self-Esteem scale; BMI, Body Mass Index; M, mean; SD, standard deviation; N, sample size; Min, minimum; Max, maximum; U, Mann-Whitney U test; t, t value; p, p value; r, correlation coefficient. ***p < .001; n.s., nonsignificant; + = one man reported this extreme but still realistic value regarding weight and BMI. In all general linear models and all generalized linear models, outlier detection marked him as an outlier and did not include him in the analyses.

For linear and generalized linear models, gender was dummy-coded, with men as the reference category. Age was centered to simplify the interpretation of the model coefficients. Due to missing data on single items within the questionnaires, the sample sizes for the initial model estimations varied, since participants were only included in the respective data analysis if they answered all items of a scale. To examine the individual impact of gender and age for each dependent variable, we started with the general linear model and inspected the residual distributions, tested statistically and by visual inspection for normality, and tested for homogeneity of variance as well as for skewness, kurtosis, and outliers (Mahalanobis and Cook’s distance, Leverage). While Cook’s distance should be smaller than 1 (70) and Leverage for large samples <3*k*/*N* (71), a value was identified as an outlier if the Mahalanobis distances were above the critical *χ* ^2^ value exceeding the probability of 0.01 (72) and if studentized deleted residuals were larger than 3 standard deviations. The highest number of outliers was detected for the Body Areas Satisfaction Scale, with 3.36%. Comparisons of the models with and without outliers revealed no substantial differences; hence, we report the models without potential outliers, as power issues were not expected for such a large sample size and precision of estimates was prioritized. Final sample sizes are reported for each model (**Tables 4** and **5**).

For the Body Areas Satisfaction Scale, the assumption of homogeneity was violated. Therefore, a general linear model was calculated, using the HC3 method for robust estimation of the standard errors. Furthermore, due to skewness and non-normal distribution of the data, responses to the Body Appreciation Scale-2 were inverted and a generalized linear model with a gamma distribution and identity link function was used. The analyses of hours people would invest in their appearance and years people would sacrifice from their lives indicated severe violations of the assumptions of the general linear model, since their distributions were similar to zero-bounded count data. Therefore, the numbers of hours and years were rounded to integer values to enable us to calculate several Poisson and negative binomial regression models, which are suitable for count data. The fit of each model was assessed by tests for overdispersion and zero inflation, as well as by tests of residual fit using the DHARMa package. As a final model for the analyses of the years people would sacrifice from their lives, we used a negative binomial regression with a log-link and linearly increasing variance (73) and adjustment for zero inflation for the intercept using the glmmTMB package. For the analyses of the hours people would spend on their appearance, we used a negative binomial regression with the log-link function using the MASS package.

## Results

### Sample Characteristics

Descriptive statistics and group differences are shown in **Table 1**. Men and women differed significantly in terms of age, height, weight, BMI, and self-esteem. Compared to women, men were slightly older, taller, and heavier and had a higher BMI. This is in line with data from the German Federal Statistical Office (57), which reported a mean weight of 68.7 kg, a mean height of 166 cm and a mean BMI of 25.1 in German women, and a mean weight of 85.0 kg, a mean height of 179 cm and a mean BMI of 26.1 in German men. As indicators of psychopathology, men and women did not differ regarding depressive mood over the past week (*p* = .152), whereas self-esteem was higher in men than in women.

Information about educational level, relationship status, number of children, and sexual orientation is reported in **Table 2**. Of the total sample, *n* = 29 participants (of whom *n* = 23 were female) refused to answer the question regarding sexual orientation, and *n* = 3 participants (of whom *n* = 1 was female) did not state whether they had children. A recent study on the proportion of Lesbian, Gay, Bisexual, and Transgender (LGBT) persons in Europe reported that 7.40% of the German population identify themselves as LGBT (74). In our sample, 10.17% reported a sexual orientation other than heterosexuality, which is slightly higher than the reported value for the German population, but can be still considered as representative.

**Table 2:** Numbers and percentages regarding educational level, relationship status, and sexual orientation for total sample, women, and men.

	Total sample (N = 1,327)		Women (N = 942)		Men (N = 385)	
	*N*	*%*	*N*	*%*	*N*	*%*
**Achieved level of education**						
No educational attainment	6	0.45	4	0.42	2	0.52
Secondary school certificate	22	1.66	9	0.96	13	3.38
General secondary or extended secondary school certificate	123	9.27	85	9.02	38	9.87
Advanced technical college certificate	88	6.63	62	6.58	26	6.75
General qualification for university entrance	451	33.99	358	38.00	93	24.16
Polytechnic degree	147	11.08	85	9.02	62	16.10
State examination/university degree	464	34.97	320	33.97	144	37.40
Other	26	1.96	19	2.02	7	1.82
**In a relationship**	836	63.0	582	61.8	254	66.0
**Sexual orientation**						
Heterosexual	1,163	87.64	820	87.05	343	89.09
Homosexual	44	3.32	23	2.44	21	5.45
Bisexual	79	5.95	66	7.01	13	3.38
Other	12	0.90	10	1.06	2	0.52
**Children**	376	28.33	246	26.11	130	33.77

N, sample size; %, percentage regarding the respective sample. In terms of sexual orientation, 29 participants (23 female) did not answer; regarding children, three participants (one female) did not answer.

The Spearman’s rank correlations of BMI with body dissatisfaction, importance of appearance, the number of hours per day participants would invest and years they would sacrifice to achieve their ideal appearance, and body appreciation are displayed in **Table 3**.

**Table 3:** Spearman’s correlations between BMI and the scores on the scales Appearance Evaluation, Body Areas Satisfaction, Appearance Orientation, the number of hours per day participants would invest to achieve their ideal appearance, and the number of years participants would sacrifice to achieve their ideal appearance and Body Appreciation for total sample, women, and men.

BMI	Appearance evaluation	Body areas satisfaction	Appearance orientation	Hours	Years	Body appreciation
**Total sample**	−.375^***^	−.344^***^	−.063^*^	.064^*^	.028	−.198^***^
*N*	1,290	1,295	1,291	1,289	1,306	1,293
						
**Women**	−.419^***^	−.399^***^	−.006	.092^**^	.074^*^	−.228^***^
*N*	915	920	919	913	927	919
						
**Men**	−.327^***^	−.318^***^	−.058	.116^*^	−.096	−.199^***^
*N*	375	375	372	376	379	374

BMI, body mass index. *p < .05; **p < .01; ***p < .001.

### General and Generalized Linear Models

**Table 4** presents the descriptive statistics for appearance evaluation, body areas satisfaction, appearance orientation, hours of investment, and years of sacrifice, as well as body appreciation, separated for total sample, men, and women. The results of the general and the generalized linear models are displayed in **Table 5**. Regarding body dissatisfaction, gender emerged as the only significant predictor of appearance evaluation (*t* = −2.012, *p* = .044) and body areas satisfaction (*t* = 4.282, *p* < .001), indicating lower appearance evaluation and lower body areas satisfaction in women than in men. Age (appearance evaluation: *t* = −1.489, *p* = .137; body areas satisfaction: *t* = −1.605, *p* = .109) and the interaction of age × gender (appearance evaluation: *t* = 1.630, *p* = .103; body areas satisfaction: *t* = 1.257, *p* = .209) did not reach statistical significance. In terms of the importance of appearance, gender (*t* = 6.597, *p* < .001), age (*t* = −3.636, *p* < .001), and the interaction of gender × age (*t* = 3.194, *p* < .001) significantly predicted appearance orientation, revealing that women placed more importance on their appearance than did men, whereas age only influenced the importance of appearance in men. The number of hours which participants would spend on their appearance if they could achieve their ideal appearance was predicted by gender (*z* = 2.037, *p* = .042) and age (*z* = −4.654, *p* < .001), indicating that women would invest more hours than men, but that with higher age, both genders would invest fewer hours in their appearance. The interaction of gender × age (*z* = 0.428, *p* = .67) was not significant. Age was the only predictor of the number of years participants would be willing to sacrifice to achieve their ideal appearance (*z* = −5.828, *p* < .001), revealing that with higher age, men and women would sacrifice fewer years for their ideal appearance. Neither gender (*z* = −0.526, *p* = .60) nor the interaction of gender × age (*z* = 1.015, *p* = .310) had a significant impact on the number of years. Furthermore, gender (*t* = 2.828, *p = .*005) and the interaction of gender × age (*t* = −2.186, *p = .*029) were significant predictors of body appreciation, insofar as with higher age, women reported higher body appreciation than men, while body appreciation in men remained stable with higher age. Age (*t* = 0.127, *p = .*899) did not reach statistical significance.

**Table 4:** Descriptive statistics regarding the scores on the scales Appearance Evaluation, Body Areas Satisfaction, Appearance Orientation, hours of investment, and years of sacrifice, as well as Body Appreciation for total sample, women and men used in the final models.

	Total sample			Women			Men		
	*M (SD)*	Min; Max	*N*	*M (SD)*	Min; Max	*N*	*M (SD)*	Min; Max	*N*
Appearance evaluation	3.49 (0.77)	1.14; 5.00	1,261	3.47 (0.79)	1.14; 5.00	913	3.55 (0.73)	1.29; 5.00	348
Body areas satisfaction	3.49 (0.64)	1.56; 5.00	1,264	3.44 (0.65)	1.56; 5.00	918	3.60 (0.59)	1.78; 5.00	346
Appearance orientation	3.11 (0.64)	1.25; 4.92	1,303	3.20 (0.62)	1.50; 4.92	929	2.91 (0.64)	1.25; 4.42	374
Hours	0.97 (1.47)	0.00; 24.00	1,294	1.04 (1.60)	0.00; 24.00	919	0.79 (1.07)	0.00; 6.00	375
Years	1.60 (3.94)	0.00; 40.00	1,317	1.64 (3.97)	0.00; 40.00	936	1.52 (3.88)	0.00; 35.00	381
Body appreciation	2.42 (0.77)	1.00; 4.90	1,306	2.46 (0.79)	1.00; 4.90	929	2.32 (0.72)	1.00; 4.80	377

M, mean; SD, standard deviation; N, sample size; Min, minimum; Max, maximum; Hours, number of hours per day men and women would invest to achieve their ideal appearance; Years, number of years men and women would sacrifice to achieve their ideal appearance.

**Table 5:** General linear models for the prediction of Appearance Evaluation, Body Areas Satisfaction and Appearance Orientation as well as generalized linear models for the prediction of Body Appreciation, the number of hours per day participants would invest to achieve their ideal appearance, and the number of years participants would sacrifice to achieve their ideal appearance, with gender and age as predictors.

	*b*	*SE (b)*	95% CI		*P*
			LL	UL	
**Appearance evaluation (** ***N*** ** = 1,261)**					
Constant	3.562	0.042	3.497	3.644	***
Gender	−0.099	0.049	−0.196	−0.002	*
Age	−0.005	0.004	−0.012	0.002	n.s.
Gender × age	0.007	0.004	−0.001	0.015	n.s.
**Body areas satisfaction (** ***N*** ** = 1,264)**					
Constant	3.444	0.022	3.402	3.487	***
Gender	−0.168	0.039	−0.244	−0.091	***
Age	−0.004	0.002	−0.009	0.001	n.s.
Gender × age	0.004	0.003	−0.002	0.010	n.s.
**Appearance orientation (** ***N*** ** = 1,303)**					
Constant	2.939	0.033	2.873	3.005	***
Gender	0.259	0.039	0.182	0.336	***
Age	−0.008	0.002	−0.012	−0.004	***
Gender × age	0.008	0.003	0.003	0.014	**
**Hours (** ***N*** ** = 1,294)**					
Constant	−0.195	0.068	−0.329	−0.064	**
Gender	0.162	0.079	0.007	0.318	*
Age	−0.024	0.005	−0.035	−0.014	***
Gender × age	0.003	0.006	−0.009	0.014	n.s.
**Years (** ***N*** ** = 1,317)**					
Constant	0.398	0.102	0.199	0.597	***
Gender	−0.058	0.110	−0.273	0.158	n.s.
Age	−0.046	0.008	−0.062	−0.031	***
Gender × age	0.0097	0.010	−0.009	0.029	n.s.
**Body appreciation (** ***N*** ** = 1,306)**					
Constant	2.315	0.039	2.241	2.394	***
Gender	0.132	0.047	0.039	0.222	**
Age	0.0003	0.003	−0.005	0.005	n.s.
Gender × age	−0.007	0.003	−0.013	−0.001	*

b, regression weights; SE(b), standard errors of the regression weights; CI, 95% confidence interval with LL, lower limit and UL, upper limit; p, p value; Hours, number of hours per day men and women would invest to achieve their ideal appearance; Years, number of years men and women would sacrifice to achieve their ideal appearance. Appearance evaluation: R^2^ = .005; body areas satisfaction: R^2^ = .013; appearance orientation: R^2^ = .052; hours: AIC = 3,418.6; years: AIC = 3,800.1; body appreciation: AIC = 2,907.8. n.s., nonsignificant; *p < .05; **p < .01; ***p < .001.

## Discussion

The aim of the present study was to investigate potential gender differences and the impact of age on body dissatisfaction, importance of appearance, the number of hours per day participants would invest and the number of years they would sacrifice to achieve their ideal appearance, and body appreciation in the general population.

As predicted in our first hypothesis, we found an effect of gender on the Appearance Evaluation Scale and the Body Areas Satisfaction Scale, suggesting that women were significantly more dissatisfied with their bodies than men. This is in accordance with the results of several studies (e.g. 28, 30, 46, 56), which likewise reported higher levels of body dissatisfaction in women than in men. In line with our results, Fallon and colleagues (29) found that women (vs. men) reported higher levels of body dissatisfaction on the Body Areas Satisfaction Scale, but contrary to our study, the authors did not find an effect of gender on the Appearance Evaluation Scale. Keel et al. (15) even found higher weight dissatisfaction in men than in women, which is also in contrast to previous findings. Therefore, it might be possible that women may be more satisfied with their weight while still reporting more body dissatisfaction.

Additionally, we found that body dissatisfaction on the Appearance Evaluation Scale and on the Body Areas Satisfaction Scale was not influenced by age or by the interaction of gender and age, indicating that body dissatisfaction remains stable across all ages for both genders. For women, this finding confirms our second hypothesis, which assumed that body dissatisfaction would not be influenced by age, and also supports previous findings (e.g. 20, 25, 29, 30). One study by Öberg and Tornstam (42) found that body satisfaction was higher in older than in younger women, which is also in contrast to our findings, as we found no influence of age on body dissatisfaction. For men, our results indicate that body dissatisfaction remains stable across different ages. This is in contrast to Ålgars and colleagues (46), who found that body dissatisfaction varied across different age groups in men. However, the latter finding might be attributable to artificial grouping strategies, as the authors investigated the impact of the continuous variable age as a categorical variable through the use of age groups. Moreover, Ålgars and colleagues (46) only assessed participants between the age of 18 and 49 years. The present study included men and women aged from 16 to 88 years, thus covering a broader proportion of the lifespan in Germany; according to the German Federal Statistical Office (75), the average life expectancy lies at 78.4 years for men and 83.2 years for women. To sum up, body dissatisfaction seems to remain relatively stable across different ages, both for men and for women.

In line with our third hypothesis that women would place more importance on their appearance than men, we found a significant effect of gender on the Appearance Orientation Scale, indicating that women indeed place more importance on their appearance compared to men. This finding corroborates previous studies (42, 50, 51). Moreover, age was a significant predictor of appearance orientation, as was the interaction of gender and age. Although age and the interaction of gender and age reached statistical significance, only in men did higher age bring about a lower importance of appearance. For women, the regression weights of age and the interaction of gender and age cancelled each other out. Therefore, gender was the only factor to impact appearance orientation in women, and the importance of appearance was not affected by age in women. This is in contrast to our fourth hypothesis that older women would report lower levels of importance of appearance than younger women. It also conflicts with previous findings (40, 41), as we found that appearance orientation remained stable across all ages in women. In line with our finding, Öberg and Tornstam (42) also reported that the importance of appearance remained stable in women of different ages. They further found a small variation of the importance of appearance across different age groups in men, with the level of importance being more pronounced from the age of 45 years and older (42). However, we observed that older men seem to place less importance on their appearance than do younger men.

As the construct of importance of appearance does not reflect the extent to which people are willing to invest time in order to reach their ideal appearance, we additionally assessed the amount of hours per day participants would invest, and the number of years of their lives they would sacrifice, in order to achieve their ideal appearance. We found an effect of gender and age on the number of hours spent on appearance, but only an effect of age on the number of years which participants would sacrifice for their appearance. Women were more likely to spend more hours per day on their ideal appearance than men. However, older men and women would invest fewer hours than their younger counterparts. Concerning the number of years people would be willing to sacrifice to achieve their ideal appearance, we found no effect of gender, but found age to be a significant predictor, meaning that older men and women would sacrifice fewer years from their lives for the sake of their ideal appearance. This indicates that in terms of their behavioral investment regarding the importance of appearance, men and women may be more similar than hitherto assumed. Apparently, women might find it easier to relinquish a small number of hours per day to be invested in their appearance compared to men, but regarding lifetime investment, both genders might be unwilling to sacrifice years of their lives for the sake of their appearance.

Furthermore, we examined the impact of gender and age on body appreciation, and found gender and the interaction of gender and age to be significant predictors. The significant effect of gender suggested that women showed less body appreciation than did men. This is in line with our fifth hypothesis that women would show lower levels of body appreciation than men, and is also in accordance with other studies (45, 53, 76). However, the significant interaction of gender and age indicates that with higher age, women report higher levels of body appreciation compared to men. This is in contrast to the aforementioned studies (e.g. 45, 53, 76), but may provide an explanation for the lack of a gender effect in an English-speaking community sample in the study by Tylka and Wood-Barcalow (55). Interestingly, compared to our study, Tylka and Wood-Barcalow (55) reported slightly higher values (from 3.22 to 3.97) for their samples for both genders. Furthermore, the significant interaction in our study suggested that body appreciation also improves in women across age, and older (vs. younger) women report higher levels of body appreciation. This is in line with Tiggemann and McCourt (20), who found greater body appreciation in older than in younger women. Regarding men, as pointed out above, no previous study has investigated the impact of age on body appreciation. In our study, the level of body appreciation remained quite stable across different ages in men, and was lower compared to that of women. An explanation might be that men are possibly more affected by restrictions of their body’s functionality due to aging processes (27), whereas women may cherish their body and the remaining functionality.

With respect to the associations between BMI and the aspects of body image, we found significant negative correlations between BMI and the Appearance Evaluation Scale and Body Areas Satisfaction Scale for men and women, insofar as with increasing BMI, values on both scales decreased (= higher body dissatisfaction). This is in line with previous research, which found that BMI was positively associated with body dissatisfaction in both genders (e.g. 77–81). Body appreciation was found to be negatively correlated with BMI for both genders, which is partially in line with previous research: One study found this association for women but not for men (53), while other studies yielded mixed findings, reporting either a negative association between BMI and body appreciation (e.g. 82, 83) or no significant results (e.g. 44). Concerning the importance of appearance, we found no significant association with BMI for either gender. In line with our results, some previous studies found no association between the importance of appearance and BMI in both men and women (13, 84), while others reported a positive correlation for women but no significant association for men (85). The latter may be explained by the differentiation between the importance of appearance and the investment of time in appearance, as we found that BMI was positively associated with the number of invested hours for both genders, but was only associated with the number of years participants would sacrifice to achieve their ideal appearance in women. These findings emphasize the distinction between the evaluative perspective of the importance of appearance (How essential are my looks to me)? and the behavioral perspective of the extent of investment in appearance (How many hours/years am I willing to invest in my appearance)?. For instance, a person may place importance on his or her appearance, but as appearance is less important than years of his or her life, he or she is unwilling to invest much effort in appearance. As shown in our study, women reported quite stable, higher levels of importance across age than did men. Consequently, it might be assumed that they have to invest more time in order to achieve their ideal appearance. Nevertheless, as older men and women would invest fewer hours and sacrifice fewer years, the extent of investment or sacrifice is evidently not expressed by the importance of appearance. These results underline the need to differentiate between the importance of appearance and the investment of time in one’s appearance.

Although in the present study, women reported a higher degree of body dissatisfaction than did men, men’s and women’s responses on average lay slightly above the value of 3 on the 5-point Likert scale (**Table 4**). This indicates, on average, neither agreement nor disagreement on the two scales (3 = I neither agree nor disagree) and possibly reveals a more neutral to slightly positive evaluation of one’s body. These results are in line with those of Cash (16) and Fallon et al. (29), who reported similar values on both scales for men and women. Therefore, on average, men and women may be neither particularly dissatisfied nor particularly satisfied with their bodies.

In consideration of all of the aforementioned research, one has to raise the more general question of whether the absence of body dissatisfaction is synonymous with the presence of body satisfaction in terms of a continuum model as proposed by Thompson et al. (4). Another possibility lies in an alternative model, in which body satisfaction and body dissatisfaction coexist alongside one another. For instance, it may be possible for a person to report high levels of overall body dissatisfaction, while simultaneously reporting high levels of body satisfaction with certain areas (e.g. “In general, I am dissatisfied with my body, but I like my legs, my cheeks and my hair.”). This could result in neither agreement nor disagreement on a continuum scale. Further research is needed to investigate a possible coexistence of both concepts.

Some limitations have to be mentioned when interpreting the results of the present study. Although several coefficients turned out to be significant, they contribute only a minimum of change to the dependent variables. In addition, according to the conventions of Cohen (86), we found very small values for the *R* ^2^s, as the *R* ^2^s in the present study explained only 0.5% (appearance evaluation) up to 5.2% (appearance orientation) of the total variance. Due to our total sample size of *N* = 1,327, the significance of the coefficients therefore might be attributed to the study’s power. Moreover, as was the case for most of the previous studies (except for 15 and 56), we did not investigate age effects in a longitudinal design. Therefore, it is not possible to disentangle the effects of age and birth cohorts. The effects found in this study may be related to different birth cohorts, the way in which people were brought up and socialized, or different ideals of beauty and fashion. Longitudinal studies including different age cohorts of men and women are therefore required.

Another limitation may lie in the assessment method. As younger people use the internet more frequently than older people (87), it cannot be excluded that this could have led to a stronger selection bias in older participants. Further, the online assessment may not be representative for the general population (88). Thus, there was no control regarding the implementation conditions of participation (e.g. whether there were distractions while participating) or regarding who was participating (88). False answers on variables such as weight, height, and age seem to be easier to notice in the laboratory. However, false statements concerning the variables of body image may be just as difficult to detect in the laboratory or in paper-and-pencil examinations as in online assessments. Our calculation of correlations between BMI and the outcome variables may be seen as a control analysis, as the participants’ answers on BMI were associated with our dependent variables, in line with aforementioned research.

Furthermore, our sample included more women than men. This may reflect the fact that women are more likely to participate in studies than men (e.g. 89, 90). Although general and generalized linear models are able to control for different sample sizes, men and women differed significantly regarding age, height, weight, and self-esteem. While the differences in weight and height could be explained by natural gender differences, men were slightly older than women. As a further limitation, the assessment was restricted to certain body-related aspects and omitted other concepts such as the drive for muscularity (35) or drive for thinness (91). We only included appearance-related aspects of body image and body appreciation in order to shorten the length of our study and to decrease the burden of our survey on respondents. Therefore, we concentrated on more general aspects related to the cognitive-affective component of body image. Future studies need to investigate the impact of gender and age on other components of body image, such as perceptual estimation of body size (e.g. 92) or checking behaviors (e.g. 93). Although some studies have already investigated body image regarding genders other than the distinct categories of male and female (e.g. 94, 95), we did not analyze these persons in the present study due to the insufficient sample size (*N* = 7). Moreover, we did not investigate the relation between sexual orientation and body image, although previous studies have found indications of an influence of sexual orientation on body image (96–99). Therefore, future research should investigate the impact of age on body image for different sexual orientations.

In conclusion, the present study is one of the first to examine body dissatisfaction, importance of appearance, the number of hours participants would be willing to invest per day to achieve their ideal appearance and the number of years they would sacrifice to achieve their ideal appearance, and body appreciation in relation to gender and age. Body appreciation was higher in older than in younger women and women reported higher levels of body appreciation compared to men. While the importance of appearance was lower in older than in younger men and remained stable in women, neither gender was willing to relinquish a large amount of time for the sake of their appearance. Although we found higher body dissatisfaction for women than for men, both genders seem to be neither satisfied nor dissatisfied with their bodies on average. Eating disorder prevention programs, or therapeutic approaches for several mental disorders, could benefit from a more functional perspective on the absence of body satisfaction, as this does not necessarily equate with the presence of body dissatisfaction.

## Data Availability Statement

The datasets generated for this study are available on request to the corresponding author.

## Ethics Statement

The studies involving human participants were reviewed and approved by Ethics committee of Osnabrück University. Written informed consent from the participants’ legal guardian/next of kin was not required to participate in this study in accordance with the national legislation and the institutional requirements.

## Author Contributions

HQ, SV, AH, and UB planned and conducted the study. RD and HQ analyzed the data. HQ wrote the first draft of the manuscript. All authors contributed to the compilation of the manuscript and read and approved the submitted version.

## Funding

We acknowledge support by Deutsche Forschungsgemeinschaft (DFG) and the Open Access Publishing Fund of Osnabrück University for the publication of the article.

## Conflict of Interest

The authors declare that the research was conducted in the absence of any commercial or financial relationships that could be construed as a potential conflict of interest.
